# CCSI: a database providing chromatin–chromatin spatial interaction information

**DOI:** 10.1093/database/bav124

**Published:** 2016-02-11

**Authors:** Xiaowei Xie, Wenbin Ma, Zhou Songyang, Zhenhua Luo, Junfeng Huang, Zhiming Dai, Yuanyan Xiong

**Affiliations:** 1 Key Laboratory of Gene Engineering of the Ministry of Education and State Key Laboratory of Biocontrol, School of Life Sciences, Sun Yat-Sen University, Guangzhou 510006, China; 2 Division of Pediatric Gastroenterology, Hepatology, and Nutrition, Department of Pediatrics, Cincinnati Children's Hospital Medical Center, University of Cincinnati, Cincinnati, OH 45229-3039, USA; 3 Department of Electronics and Communication Engineering, School of Information Science and Technology, Sun Yat-Sen University, Guangzhou 510006, China; 4 SYSU-CMU Shunde International Joint Research Institute, Shunde, China

## Abstract

Distal regulatory elements have been shown to regulate gene transcription through spatial interactions, and single nucleotide polymorphisms (SNPs) are linked with distal gene expression by spatial proximity, which helps to explain the causal role of disease-associated SNPs in non-coding region. Therefore, studies on spatial interactions between chromatin have created a new avenue for elucidating the mechanism of transcriptional regulation in disease pathogenesis. Recently, a growing number of chromatin interactions have been revealed by means of 3C, 4C, 5C, ChIA-PET and Hi-C technologies. To interpret and utilize these interactions, we constructed chromatin–chromatin spatial interaction (CCSI) database by integrating and annotating 91 sets of chromatin interaction data derived from published literature, UCSC database and NCBI GEO database, resulting in a total of 3 017 962 pairwise interactions (false discovery rate < 0.05), covering human, mouse and yeast. A web interface has been designed to provide access to the chromatin interactions. The main features of CCSI are (i) showing chromatin interactions and corresponding genes, enhancers and SNPs within the regions in the search page; (ii) offering complete interaction datasets, enhancer and SNP information in the download page; and (iii) providing analysis pipeline for the annotation of interaction data. In conclusion, CCSI will facilitate exploring transcriptional regulatory mechanism in disease pathogenesis associated with spatial interactions among genes, regulatory regions and SNPs.

**Database URL**: http://120.79.23.67/ccsi/search.php

## Introduction

Chromatin folds and forms spatial structure inside cell nucleus instead of linear stretching (1). Spatial proximity between chromatin segments may result from direct physical contact or indirect co-localization mediated by particular protein or stochastic collision in the congested nucleus (2). However, traditional experimental methods applied to capture the three-dimensional (3D) organization of chromosome such as microscope and fluorescence *in situ* hybridization (FISH) are technically challenging or have low-throughput limitation (1, 3).

During the past decade, several approaches that combined traditional experimental methods and cutting-edge sequencing technologies have contributed much to exploring the spatial structure of chromosome at both higher resolution and throughput. These approaches are based on coupling ligation in close spatial proximity, followed by high-throughput sequencing (2). Chromosome conformation capture (3C) captures one-to-one interaction at a time while circular 3C or 3C-on-chip (4C) produces a genome-wide interaction profile for a given locus (1, 3). Chromosome conformation capture carbon copy (5C) and Hi-C (including capture Hi-C) generate multiple pairs of interactions in an unbiased way rather than focus on a single locus (4, 5). Chromatin interaction analysis with paired-end-Tag (ChIA–PET) enables to observe interactions between loci bound by specific protein in whole genome (6).

Furthermore, spatial contacts between chromatin have implicated functional roles in transcriptional regulation (3, 7). For example, enhancers up-regulate gene expression by associating with their targets (8). Inactive genes tend to be spatially co-localized with regions marked by repressive histone modification such as H3K9me3 and H3K27me3 (9–11). Genes in spatial proximity are likely to be co-regulated and functionally related (12). Besides, single nucleotide polymorphisms (SNPs) have been shown to connect with the expression of far-away genes through spatial proximity (13). Moreover, a classical example is obesity-associated SNP (rs9930506), which gives rise to obesity by regulating the expression of *IRX3* through long-range connection (14). These demonstrate that the higher-order structure of chromatin plays critical roles in the regulation of gene expression.

For a better understanding of chromatin interaction, transcriptional regulation and disease, we present a chromatin–chromatin spatial interaction (CCSI) database that integrates 91 sets of published chromatin interaction data, contains 3 017 962 high confident interaction pairs [false discovery rate (FDR) < 0.05], and covers three species: human, mouse and yeast. CCSI has three characteristics: (i) a user-friendly search page for the comprehensive retrieval of chromatin interactions, (ii) a straightforward download page with annotated interaction datasets and unannotated interaction pairs for the location of other interested regions and (iii) open source code for the annotation of interaction data in the GitHub (http://120.79.23.67/ccsi/search.php). These resources are particularly valuable for discovering molecular regulatory mechanisms involving spatial interactions.

## Methods

### Data sources

A total of 91 datasets of chromatin interactions from three species were collected from National Center for Biotechnology Information (NCBI), Gene Expression Omnibus (GEO), database, University of California Santa Cruz (UCSC) database or the supplementary data of literature (Supplementary Table S1). With regard to each dataset, we counted the median length of all chromatin fragments as its interaction resolution. Together with data sources, they were summarized in Supplementary Table S1. The reference genome annotations of human (UCSC hg38) and mouse (UCSC mm10) were obtained from GENCODE database (15). The genome annotations of *Schizosaccharomyces pombe* and *Saccharomyces cerevisiae* were downloaded from Ensembl and Saccharomyces Genome Database (SGD) database, respectively. SNPs (hg38-db141 and mm10-db137) were downloaded from UCSC database (16, 17).

### Annotation of interaction data

**Figure 1:**
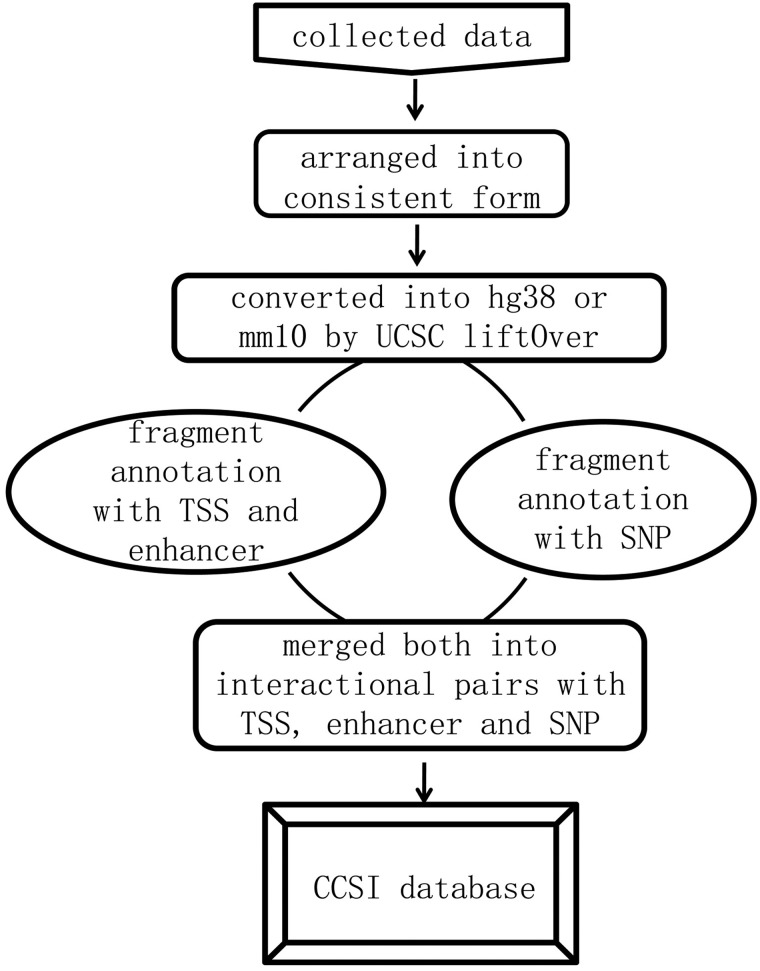
Framework of constructing CCSI database.

We first formatted the interaction data into ‘frag_1, frag_2, contact, FDR and *P*-value’. Frag_1 and frag_2 were the interaction pair, each represented by a genomic region: chromosome: start-end. Contact was the raw read counts of interaction pair and we assigned ‘NA’ to the missing value. FDR and *P*-value were provided for assessing the significance of contact frequency. They came directly from original literature or were equal to expected interactions divided by observed interactions (18, 19), and each of which could be NA for the missing value. All interaction data were converted into the current version (hg38 and mm10) by UCSC liftOver prior to annotation. Then the location of transcription start site (TSS), midpoint of enhancer and SNP in specific frag_1 or frag_2 was determined by bedtools (intersectBed) (20). For each promoter, we identified the fragment covering its TSS. For each enhancer region, we identified the fragment covering its midpoint to guarantee at least 50% regional overlap between them. Eventually, the data format was transformed into chromatin interaction pair with the annotation of promoters, enhancers and SNPs, and summarizing the information for it. The analysis pipeline for annotation and corresponding instruction were released in the GitHub. The whole process was shown in Figure 1.

## Results

### Website contents

**Figure 2:**
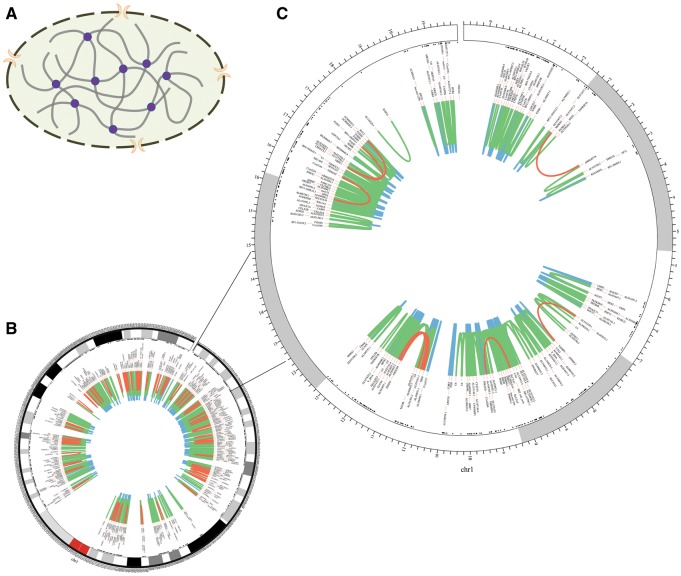
Chromosomal 3D structure and promoter–promoter interactions of Chr1 in IMR90 cell line based on a set of Hi-C data. (**A**) Chromosomal 3D structure. The dashed circle with two orange crescents that stand for nuclear pore complex is the nucleus membrane. The thick grey lines are chromatin and the purple circles stand for proteins that link chromatin together. (**B**) Promoter–promoter interactions of Chr1. (**C**) Promoter–promoter interactions of Chr1:1-20000000, zooming into the interactions. The red lines stand for long-range interactions (distance between interaction pair > 500 kb), while the blue lines for short-range (distance < 50 kb) and the green lines for middle-range (distance spanning 50–500 kb). The black texts are the gene names of corresponding loci.

**Figure 3:**
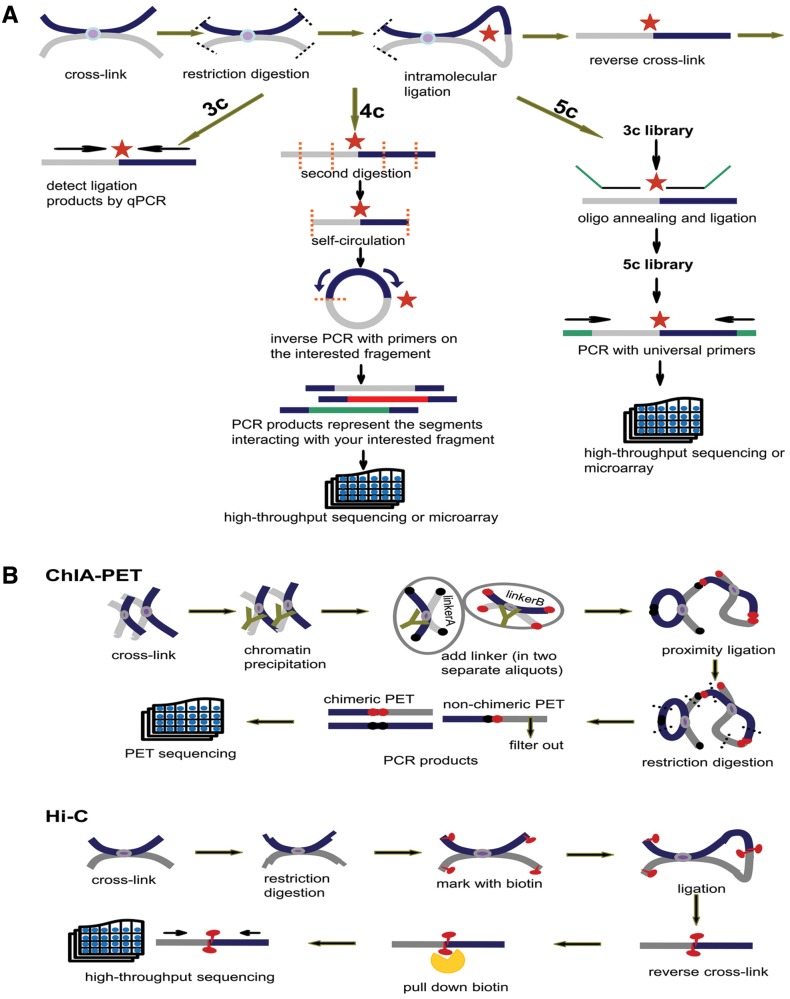
Principles of 3C, 4C, 5C, ChIA-PET and Hi-C. (**A**) Principles of 3C, 4C and 5C. The thick dark blue and grey lines stand for two different chromatin fragments and the purple dot in the middle is the protein that binds them together. Both black and orange dashed lines represent the restriction sites and the red star is the ligation site of two fragments. The arrows parallel with chromatin fragments represent primers. The green line in 5C is the oligo sequence. (**B**) Principles of ChIA-PET and Hi-C. The notations appearing in (A) have the same meaning. In ChIA-PET pipeline the tawny ‘Y’ shape represents the antibody specifically binding to the protein (purple dot). The black and dark red dots stand for linkerA and linkerB, respectively. Finally, in Hi-C pipeline the red tadpole is biotin mark, which will be pulled down by streptavidin bead (orange scissor).

The homepage displays a brief 3D structure of chromosome and an example of promoter–promoter interaction pairs generated by Circos (21) (Figure 2). Principles of 3C-based approaches are presented in the method page (Figure 3). Search and download pages are provided to query and acquire spatial interactions.

Chromatin segments fold and contact with each other in the cell nucleus, which creates opportunity for functional relationship between them (Figure 2A). Long-range interactions occur less frequently than median- and short-range ones (Figure 2B and C). Moreover, genes within the same topologically associating domains (TADs) interact more frequently than those from different TADs (Figure 2C) (10, 18).

Schematic representations of 3C, 4C, 5C, ChIA-PET and Hi-C technologies are introduced in the method page. The initial steps of 3C, 4C and 5C are identical: (i) covalent fixation of chromatin in close spatial proximity by formaldehyde, (ii) digestion of cross-linked chromatin with restriction enzyme, (iii) ligation of hybrid DNA molecule, (iiii) reverse linking of chimeric DNA molecule. Then, relying on qPCR with locus-specific primers, 3C detects a pair of interaction. With the second digestion on ligation product, 4C generates a genomic interaction profile around a single locus by way of inverse PCR. 5C identifies multiple pairs of interactions with universal primers after the ligation of oligo sequence (Figure 3A). Based on chromatin immunoprecipitation and paired-end tag sequencing, ChIA-PET allows for genome-wide analysis of interactions bound by specific protein. Hi-C unbiasedly unveils interactions of any two loci in the whole genome. The chimeric fragments are marked with biotin, which could be pulled down with streptavidin beads and sequenced in high-throughput (Figure 3B).

Search and download pages are designed for providing users with comprehensive interaction information. Search page enables users to inquire chromatin fragments, genes, enhancers and SNPs that spatially interact with genes or regions of interests. Annotated interaction datasets together with unannotated interaction pairs are also available in the download page. All chromatin interaction data supplied in CCSI are with a threshold of FDR < 0.05.

### Search engine

The main purpose of CCSI is to support search for chromatin fragments, genes, enhancers and SNPs that spatially contact with genes or regions of interests. First, one or more of methods (e.g. 3C, 4C, etc.), species and cell types can be submitted to constrain search range. Otherwise, by default, CCSI will report chromatin interaction pairs with the annotation of genes, enhancers and SNPs from three species through 3C, 4C, 5C, ChIA-PET and Hi-C (including CHi-C) technologies. The search engine is primarily designed to receive two types of queries: (i) Ensembl ID like ENSG00000230368 or official gene symbol like FAM41C; and (ii) chromatin fragment like chr1:1-1000. With regard to unextended search results, clicking ‘plus’ button will give details. Chromatin fragments were linked to a genome browser page by using the JBrowse (JBrowse-1.11.6) (22). SNPs can be represented by chromatin fragments; hence, distal genes that spatially associate with genome wide association studies (GWAS) SNPs are also available.

#### Example of CCSI search

**Figure 4:**
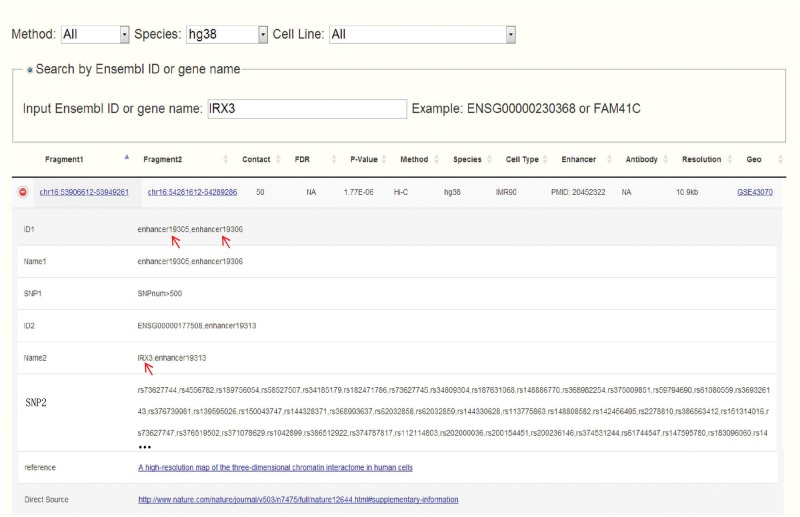
Search result for human after inputting *IRX3*. Chr16:53906612-53949261 which contains enhancers and overlaps with the intron of *FTO* spatially interacts with chr16:54281612-54289286 which contains the promoter of *IRX3*.

Obesity-associated *FTO* intron has been reported to contain enhancers and spatially connect with *IRX3* in both human and mouse (14). Set *IRX3* as input, CCSI gives a list of chromatin interaction pairs involving *IRX3*. Among the outputs for human, chr16:53906612-53949261 that spatially associates with *IRX3* contains enhancers and also overlaps with the intron of *FTO* (Figure 4), which coincide with (14). While for mouse, partial result like the interaction between proximal enhancer and *IRX3* presents the strong interaction signal of (14). A possible reason for missing *IRX3*-*FTO* pair in mouse is that a weak interaction is filtered out. In summary, CCSI presents users with abundant chromatin interaction information, which would provide powerful support for biomedical research.

### Download page

It is convenient to acquire the chromatin interactions of interests in the search page. Meanwhile, we provide complete interaction datasets in the download page, facilitating for the comprehensive analysis based on massive data. Furthermore, genome annotation files, enhancer region files and dbSNPs are also available for download.

## Discussion

In this study, we developed CCSI database for the collection, interpretation and analysis of chromatin interaction data. CCSI is a database providing users with a multitude of interaction pairs in human, mouse and yeast. The most remarkable feature for information acquisition is search engine, which permits Ensembl ID, gene name or chromatin fragment as input, and presents accurate and reliable interaction information.

It has been shown that genes that spatially contact with larger numbers of distal regulatory elements (DREs) are more likely to have essential functions (12). Moreover, genes in the same TADs tend to be co-regulated and functionally related (2, 12, 23). Meanwhile, target genes of transcription factors are co-localized in the nucleus (24). Both of them suggest that genes would compose functional 3D cluster corresponding to specific biological pathway. Moreover, functional region compartmentalization should be referred to 3D data instead of linear level data. Abundant interaction pairs in CCSI would contribute to a deeper excavation of the regulatory mechanism concerning chromatin interaction network.

Accumulating SNPs have been linked to the expression of distal genes through spatial contacts (13). For instance, SNP in *FTO* intron causes obesity by long-range functional connection with *IRX3*, which testifies the causal role of disease-associated SNPs in non-coding region (14). Besides, CHi-C data have facilitated predicting and analysing the potential targets of breast cancer susceptibility loci in an unbiased way (25). Recently, key long-range chromatin interactions involving 14 colorectal cancer risk loci have been identified in virtue of CHi-C (26). These pave the way for exploring the mechanisms underscoring GWAS signals for complex diseases. Here, CCSI enables users to obtain spatial targets of SNPs, which would give valuable insights into the research of disease pathogenesis.

We anticipate continuous updates of CCSI as more chromatin interaction experiments are being conducted in a wide range of cell types and species. We will continuously keep our eyes on the progress about chromatin interactions. With the help of a flexible analysis pipeline, CCSI will continue to serve as an important resource with timely update of the newest chromatin interaction data. In addition, mutation data from The Cancer Genome Atlas (TCGA) and epigenetic data from Roadmap Epigenomics Consortium will also be integrated into CCSI in our next version, achieving more comprehensive understanding of chromatin interaction network and its role in understanding the development and progression of diseases.

## Supplementary Data

Supplementary data are available at *Database* Online.

## Supplementary Material

Supplementary Data
