# Supplementary material for: CCSI: a database providing chromatin–chromatin spatial interaction information
Source: Database (Oxford). 2016 Feb 11;2016:bav124. doi: 10.1093/database/bav124 (PMC4750547; doi:10.1093/database/bav124)
Supplement: Supplementary Data [file bav124_supplementary_data.zip › Supplementary_data.doc]

**Supplementary data**

CCSI: a database providing Chromatin-Chromatin Spatial Interaction information

**Supplementary Table 1 Chromatin interaction datasets used in this study**

| **Method** | **Original Assembly** | **Cell Type** | **Enhancer Source** | **Antibody** | **GEO Number** | **Data Source** | **Reference** | **Resolution** |
| --- | --- | --- | --- | --- | --- | --- | --- | --- |
| 3C | fission yeast | NA | NA | NA | SRP002804 | PubMed | (1) | 20.0kb |
| 4 C | mm6 | fetal liver | NA | β-globin | GSE5891 | PubMed | (2) | 31.2kb |
| 4 C | mm6 | fetal brain | NA | β-globin | GSE5891 | PubMed | (2) | 29.6kb |
| 4 C | mm6 | fetal liver | NA | Rad23a | GSE5891 | PubMed | (2) | 18.7kb |
| 4 C | mm6 | fetal brain | NA | Rad23a | GSE5891 | PubMed | (2) | 18.1kb |
| 4 C | mm9 | mESC | PMID: 21632746 | Oct4 | GSE45418 | PubMed | (3) | 95.8kb |
| 4 C | hg18 | K562 | FANTOM5 | CNC,nonCNC | NA | PubMed | (4) | 0.2kb |
| 4 C | hg18 | GM06990 | NA | LCR-HS5 | NA | PubMed | (4) | 1.8kb |
| 4 C | saccer2 | NA | NA | NA | SRP002120 | PubMed | (5) | 1.0kb |
| 4 C | saccer2 | NA | NA | NA | SRP002120 | PubMed | (5) | 1.0kb |
| 5 C | hg18 | GM12878 | FANTOM5 | α-globin | NA | PubMed | (6) | 5.2kb |
| 5 C | hg18 | K562 | FANTOM5 | α-globin | NA | PubMed | (6) | 5.5kb |
| 5 C | mm9 | MEF-female | PMID: 22763441 | NA | GSE35721 | Pubmed | (7) | 2.0kb |
| 5 C | mm9 | MEF-male | PMID: 22763441 | NA | GSE35721 | PubMed | (7) | 2.2kb |
| 5 C | mm9 | mESCs-female-PGK12.1-day2 | PMID: 21632746 | NA | GSE35721 | PubMed | (7) | 2.2kb |
| 5 C | mm9 | mESCs-female-PGK12.1-undiff | PMID: 21632746 | NA | GSE35721 | PubMed | (7) | 2.2kb |
| 5 C | mm9 | mESCs-femaleXO-DXTX-undiff | PMID: 21632746 | NA | GSE35721 | PubMed | (7) | 2.0kb |
| 5 C | mm9 | mESCs-male-E14 | PMID: 21632746 | NA | GSE35721 | PubMed | (7) | 2.2kb |
| 5 C | mm9 | mESCs-male-E14-undiff | PMID: 21632746 | NA | GSE35721 | PubMed | (7) | 2.1kb |
| 5 C | mm9 | mESCs-male-EED-undiff | PMID: 21632746 | NA | GSE35721 | PubMed | (7) | 1.9kb |
| 5 C | mm9 | mESCs-male-TT2G9A-undiff | PMID: 21632746 | NA | GSE35721 | PubMed | (7) | 2.1kb |
| 5 C | mm9 | mESCs-male-TT2-undiff | PMID: 21632746 | NA | GSE35721 | PubMed | (7) | 2.0kb |
| 5 C | mm9 | mNPCs-male-E14 | PMID: 21632746 | NA | GSE35721 | PubMed | (7) | 2.1kb |
| 5 C | hg19 | BJ | NA | NA | GSE39505 | ENCODE (UW) | NA | 4.7kb |
| 5 C | hg19 | Caco2 | NA | NA | GSE39505 | ENCODE (UW) | NA | 4.1kb |
| 5 C | hg19 | GM06990 | NA | NA | GSE39505 | ENCODE (UW) | NA | 4.7kb |
| 5 C | hg19 | GM12878 | FANTOM5 | NA | GSE39505 | ENCODE (UW) | NA | 4.2kb |
| 5 C | hg19 | HepG2 | UCSC-H3k27ac | NA | GSE39505 | ENCODE (UW) | NA | 4.5kb |
| 5 C | hg19 | K562 | FANTOM5 | NA | GSE39505 | ENCODE (UW) | NA | 4.4kb |
| 5 C | hg19 | LNCaP | NA | NA | GSE39505 | ENCODE (UW) | NA | 4.2kb |
| 5 C | hg19 | Mcf7 | UCSC-H3k27ac | NA | GSE39505 | ENCODE (UW) | NA | 4.2kb |
| 5 C | hg19 | SK-N-SH | NA | NA | GSE39505 | ENCODE (UW) | NA | 4.9kb |
| 5 C | hg19 | HelaS3 | FANTOM5 | NA | GSE39510 | PubMed | (8) | 4.6kb |
| 5 C | hg19 | hESC | PMID: 20452322 | NA | GSE39510 | PubMed | (8) | 4.7kb |
| 5 C | hg19 | GM12878 | FANTOM5 | NA | GSE39510 | PubMed | (8) | 4.9kb |
| 5 C | hg19 | K562 | FANTOM5 | NA | GSE39510 | PubMed | (8) | 5.0kb |
| ChIA-PET | hg18 | Mcf7 | UCSC-H3k27ac | ER-α | GSE18046 | PubMed | (9) | 5.5kb |
| ChIA-PET | hg19 | K562 | FANTOM5 | RNAPII | GSE33664 | PubMed | (10) | 8.8kb |
| ChIA-PET | hg19 | Mcf7 | UCSC-H3k27ac | RNAPII | GSE33664 | PubMed | (10) | 7.7kb |
| ChIA-PET | hg18 | CD4^+^ T cell | NA | H3K4me2 | GSE32677 | PubMed | (11) | 6.7kb |
| ChIA-PET | hg19 | Hct116 | UCSC-H3k27ac | RNAPII | GSE39495 | ENCODE (GIS-ruan) | NA | 0.7kb |
| ChIA-PET | hg19 | NB4 | NA | RNAPII | GSE39495 | ENCODE (GIS-ruan) | NA | 0.7kb |
| ChIA-PET | hg19 | HelaS3 | FANTOM5 | RNAPII | GSE39495 | ENCODE (GIS-ruan) | NA | 0.7kb |
| ChIA-PET | hg19 | K562 | FANTOM5 | RNAPII | GSE39495 | ENCODE (GIS-ruan) | NA | 2.4kb |
| ChIA-PET | hg19 | Mcf7 | UCSC-H3k27ac | RNAPII | GSE39495 | ENCODE (GIS-ruan) | NA | 2.4kb |
| ChIA-PET | hg19 | Mcf7 | UCSC-H3k27ac | ER-α | GSE39495 | ENCODE (GIS-ruan) | NA | 0.8kb |
| ChIA-PET | hg19 | Mcf7 | UCSC-H3k27ac | CTCF | GSE39495 | ENCODE (GIS-ruan) | NA | 0.8kb |
| ChIA-PET | hg19 | K562 | FANTOM5 | CTCF | GSE39495 | ENCODE (GIS-ruan) | NA | 0.8kb |
| ChIA-PET | mm9 | mESC | PMID: 21632746 | RNAPII | GSE44067 | PubMed | (12) | 3.0kb |
| ChIA-PET | mm9 | mNSC | NA | RNAPII | GSE44067 | PubMed | (12) | 5.5kb |
| ChIA-PET | mm9 | mNPC | PMID: 21632746 | RNAPII | GSE44067 | PubMed | (12) | 3.7kb |
| ChIA-PET | mm9 | e11.5limb | PMID: 22763441 | Smc1a | GSE42237 | PubMed | (13) | 4.0kb |
| Hi-C | mm9 | cerebellum | PMID: 22763441 | NA | GSE34587 | PubMed | (14) | 2.5kb |
| Hi-C | mm9 | cortex | PMID: 22763441 | NA | GSE34587 | PubMed | (14) | 2.5kb |
| Hi-C | mm9 | E14.5-brain | PMID: 22763441 | NA | GSE34587 | PubMed | (14) | 2.5kb |
| Hi-C | mm9 | E14.5-heart | PMID: 22763441 | NA | GSE34587 | PubMed | (14) | 3.0kb |
| Hi-C | mm9 | E14.5-limb | PMID: 22763441 | NA | GSE34587 | PubMed | (14) | 3.0kb |
| Hi-C | mm9 | heart | PMID: 22763441 | NA | GSE34587 | PubMed | (14) | 2.5kb |
| Hi-C | mm9 | intestine | PMID: 22763441 | NA | GSE34587 | PubMed | (14) | 2.6kb |
| Hi-C | mm9 | kidney | PMID: 22763441 | NA | GSE34587 | PubMed | (14) | 2.5kb |
| Hi-C | mm9 | liver | PMID: 22763441 | NA | GSE34587 | PubMed | (14) | 3.0kb |
| Hi-C | mm9 | lung | PMID: 22763441 | NA | GSE34587 | PubMed | (14) | 2.5kb |
| Hi-C | mm9 | MEF | PMID: 22763441 | NA | GSE34587 | PubMed | (14) | 3.0kb |
| Hi-C | mm9 | mESC | PMID: 22763441 | NA | GSE34587 | PubMed | (14) | 2.7kb |
| Hi-C | mm9 | olfactory | PMID: 22763441 | NA | GSE34587 | PubMed | (14) | 2.5kb |
| Hi-C | mm9 | placenta | PMID: 22763441 | NA | GSE34587 | PubMed | (14) | 3.0kb |
| Hi-C | mm9 | spleen | PMID: 22763441 | NA | GSE34587 | PubMed | (14) | 2.5kb |
| Hi-C | mm9 | testes | PMID: 22763441 | NA | GSE34587 | PubMed | (14) | 2.5kb |
| Hi-C | mm9 | thymus | PMID: 22763441 | NA | GSE34587 | PubMed | (14) | 3.0kb |
| Hi-C | mm9 | mESC | PMID: 21632746 | NA | GSE35156 | PubMed | (15) | 40.0kb |
| Hi-C | mm9 | cortex | PMID: 22763441 | NA | GSE35156 | PubMed | (15) | 40.0kb |
| Hi-C | hg18 | hESC | PMID: 20452322 | NA | GSE35156 | PubMed | (15) | 40.0kb |
| Hi-C | hg18 | IMR90 | PMID: 20452322 | NA | GSE35156 | PubMed | (15) | 40.0kb |
| Hi-C | hg18 | IMR90 | PMID: 20452322 | NA | GSE43070 | PubMed | (16) | 10.9kb |
| situ Hi-C | hg19 | GM12878 | FANTOM5 | NA | GSE63525 | PubMed | (17) | 10.0kb |
| situ Hi-C | hg19 | HelaS3 | FANTOM5 | NA | GSE63525 | PubMed | (17) | 25.0kb |
| situ Hi-C | hg19 | HMEC | NA | NA | GSE63525 | PubMed | (17) | 10.0kb |
| situ Hi-C | hg19 | HUVEC | NA | NA | GSE63525 | PubMed | (17) | 25.0kb |
| situ Hi-C | hg19 | IMR90 | PMID: 20452322 | NA | GSE63525 | PubMed | (17) | 10.0kb |
| situ Hi-C | hg19 | K562 | FANTOM5 | NA | GSE63525 | PubMed | (17) | 10.0kb |
| situ Hi-C | hg19 | KBM7 | NA | NA | GSE63525 | PubMed | (17) | 25.0kb |
| situ Hi-C | mm9 | mouse_lymphoblasts | NA | NA | GSE63525 | PubMed | (17) | 10.0kb |
| situ Hi-C | hg19 | NHEK | NA | NA | GSE63525 | PubMed | (17) | 25.0kb |
| CHi-C | hg19 | GM12878 | NA | NA | NA | PubMed | (18) | 7.3kb |
| CHi-C | hg19 | GM12878 | FANTOM5 | NA | NA | PubMed | (18) | 5.2kb |
| CHi-C | hg19 | CD34 | NA | NA | NA | PubMed | (18) | 7.6kb |
| CHi-C | hg19 | CD34 | NA | NA | NA | PubMed | (18) | 5.9kb |
| CHi-C | mm9 | mESC | NA | NA | NA | PubMed | (19) | 10.0kb |
| CHi-C | mm9 | mESC | NA | NA | NA | PubMed | (19) | 4.7kb |
| CHi-C | mm9 | fetal liver | PMID: 21632746 | NA | NA | PubMed | (19) | 10.0kb |
| CHi-C | mm9 | fetal liver | NA | NA | NA | PubMed | (19) | 4.1kb |

**References**

1. Tanizawa, H., Iwasaki, O., Tanaka, A.*, et al.* (2010) Mapping of long-range associations throughout the fission yeast genome reveals global genome organization linked to transcriptional regulation. *Nucleic Acids Res*, **38**, 8164-8177.

2. Simonis, M., Klous, P., Splinter, E.*, et al.* (2006) Nuclear organization of active and inactive chromatin domains uncovered by chromosome conformation capture-on-chip (4C). *Nat Genet*, **38**, 1348-1354.

3. Wei, Z., Gao, F., Kim, S.*, et al.* (2013) Klf4 organizes long-range chromosomal interactions with the oct4 locus in reprogramming and pluripotency. *Cell Stem Cell*, **13**, 36-47.

4. Robyr, D., Friedli, M., Gehrig, C.*, et al.* (2011) Chromosome Conformation Capture Uncovers Potential Genome-Wide Interactions between Human Conserved Non-Coding Sequences. *Plos One*, **6**.

5. Duan, Z., Andronescu, M., Schutz, K.*, et al.* (2010) A three-dimensional model of the yeast genome. *Nature*, **465**, 363-367.

6. Bau, D., Sanyal, A., Lajoie, B.R.*, et al.* (2011) The three-dimensional folding of the alpha-globin gene domain reveals formation of chromatin globules. *Nat Struct Mol Biol*, **18**, 107-114.

7. Nora, E.P., Lajoie, B.R., Schulz, E.G.*, et al.* (2012) Spatial partitioning of the regulatory landscape of the X-inactivation centre. *Nature*, **485**, 381-385.

8. Sanyal, A., Lajoie, B.R., Jain, G.*, et al.* (2012) The long-range interaction landscape of gene promoters. *Nature*, **489**, 109-113.

9. Fullwood, M.J., Liu, M.H., Pan, Y.F.*, et al.* (2009) An oestrogen-receptor-alpha-bound human chromatin interactome. *Nature*, **462**, 58-64.

10. Li, G., Ruan, X., Auerbach, R.K.*, et al.* (2012) Extensive promoter-centered chromatin interactions provide a topological basis for transcription regulation. *Cell*, **148**, 84-98.

11. Chepelev, I., Wei, G., Wangsa, D.*, et al.* (2012) Characterization of genome-wide enhancer-promoter interactions reveals co-expression of interacting genes and modes of higher order chromatin organization. *Cell Res*, **22**, 490-503.

12. Zhang, Y., Wong, C.H., Birnbaum, R.Y.*, et al.* (2013) Chromatin connectivity maps reveal dynamic promoter-enhancer long-range associations. *Nature*, **504**, 306-310.

13. DeMare, L.E., Leng, J., Cotney, J.*, et al.* (2013) The genomic landscape of cohesin-associated chromatin interactions. *Genome Res*, **23**, 1224-1234.

14. Shen, Y., Yue, F., McCleary, D.F.*, et al.* (2012) A map of the cis-regulatory sequences in the mouse genome. *Nature*, **488**, 116-120.

15. Dixon, J.R., Selvaraj, S., Yue, F.*, et al.* (2012) Topological domains in mammalian genomes identified by analysis of chromatin interactions. *Nature*, **485**, 376-380.

16. Jin, F., Li, Y., Dixon, J.R.*, et al.* (2013) A high-resolution map of the three-dimensional chromatin interactome in human cells. *Nature*, **503**, 290-294.

17. Rao, S.S.P., Huntley, M.H., Durand, N.C.*, et al.* (2014) A 3D Map of the Human Genome at Kilobase Resolution Reveals Principles of Chromatin Looping. *Cell*, **159**, 1665-1680.

18. Mifsud, B., Tavares-Cadete, F., Young, A.N.*, et al.* (2015) Mapping long-range promoter contacts in human cells with high-resolution capture Hi-C. *Nat Genet*, **47**, 598-606.

19. Schoenfelder, S., Furlan-Magaril, M., Mifsud, B.*, et al.* (2015) The pluripotent regulatory circuitry connecting promoters to their long-range interacting elements. *Genome Res*, **25**, 582-597.
